# Salmonid fish: model organisms to study cardiovascular morphogenesis in conjoined twins?

**DOI:** 10.1186/s12861-016-0125-x

**Published:** 2016-07-16

**Authors:** Per Gunnar Fjelldal, Monica F. Solberg, Tom Hansen, Tone Vågseth, Kevin Alan Glover, Harald Kryvi

**Affiliations:** Institute of Marine research (IMR), Matre Aquaculture Research Station, Matredal, Norway; Institute of Marine research (IMR), Bergen, Norway; Department of Biology, University of Bergen, Bergen, Norway; Havforskningsinstituttet, Matre, Matre 5, 5984 Matredal, Norway

**Keywords:** Blood flow, Morphogenesis, Respiration, Blood vessels

## Abstract

**Background:**

There is a gap in knowledge regarding the cardiovascular system in fish conjoined twins, and regarding the cardiovascular morphogenesis of conjoined twins in general. We examined the cardiovascular system in a pair of fully developed ventrally conjoined salmonid twins (45.5 g body weight), and the arrangement of the blood vessels during early development in ventrally conjoined yolk sac larvae salmonid twins (<0.5 g body weight).

**Results:**

In the fully developed twins, one twin was normal, while the other was small and severely malformed. The mouth of the small twin was blocked, inhibiting respiration and feeding. Both twins had hearts, but these were connected through a common circulatory system. They were joined by the following blood vessels: (i) *arteria iliaca* running from *arteria caudalis* of the large twin to the kidney of the small twin; (ii) *arteria subclavia* running from *aorta dorsalis* of the large twin to *aorta dorsalis* of the small twin; (iii) *vena hepatica* running from the liver of the small twin into the *sinus venosus* of the large twin. Among the yolk sac larvae twins investigated, distinct vascular connections were found in some individuals through a joined *v. vitellina hepatica*.

**Conclusions:**

Ventrally conjoined fish twins can develop cardiovascular connections during early development, enabling a normal superior twin to supply a malfunctioning twin with oxygen and nutrients. Since the yolk sac in salmonids is transparent, twinning in salmonids may be a useful model in which to study cardiovascular morphogenesis in conjoined twins.

**Electronic supplementary material:**

The online version of this article (doi:10.1186/s12861-016-0125-x) contains supplementary material, which is available to authorized users.

## Background

Conjoined twins are frequently observed in hatcheries for a range of different fish species, and several different phenotypes have been observed; mostly duplets, more rarely triplets. The phenomenon of conjoined twining in fish has been broadly studied, and the first record dates back to the 17^th^ century (reviewed in [1]). It was initially suggested that the conjoined condition could only be explained by the fusion of already separate individuals and subsequent resorption of already formed parts based on a study on the internal anatomy of about 400 conjoined salmonid twin embryos (reviewed in [1]). Later, it has been shown that a primary fission of the early cleaving blastoderm can result in the formation of two adjacent blastoderms that undergo secondary fusion during epiboly, resulting in conjoined twins [2]. Conjoined fish twins rarely achieve adulthood, and typically die during the first feeding period. However, there are reports on conjoined fish twins that reach adulthood; a 1.7 kg wild caught rainbow trout (*Oncorhynchus mykiss*) [3], a 3.5 kg farmed Atlantic salmon (*Salmo salar*) [4], a 30.5 cm long farmed hybrid catfish (*Ictalurus punct*-*atus* ♀ x *Ictalurus furcatus* ♂) [5], a 1.9 cm long Endlers guppy (*Poecilia wingei*) [6], all with a parasitic twin grown into the body wall. In the hybrid catfish [5], the parasitic twin was mostly vestigial, consisting of partial elements of the musculoskeletal, gastrointestinal and central nervous systems, while the normal twin appeared to be viable and showed no signs of pathology based on histology, gross examination, and radiology. Although such parasitic fish twins most probably must depend on nutrients and oxygen supplied by their normal sibling, the cardiovascular system of conjoined fish twins has never been studied in detail.

In contract to fish, the cardiovascular system of human conjoined twins has been investigated. These studies have shown that human conjoined twins can have no union in cardiac, aortic, and inferior vena caval level, or have a union with either separate hearts or one common heart [7–11]. Nonetheless, there is a gap in knowledge regarding the cardiovascular morphogenesis of conjoined twins in general.

In the present study, we investigated a conjoined twin specimen detected in a first-generation hybrid between an Atlantic salmon (♀) and an Arctic char (*Salvelinus alpines*) (♂) that was raised at the Institute of Marine Research, Matre Research Station. The twins were 1 year old and ventrally connected by their abdomens. A detailed study of macro anatomy and tissue histology, with special emphasis on their cardiovascular systems, was performed. The yolk sac of the salmonid alevin is transparent, allowing a direct view of the cardiovascular system [12, 13]. Indeed, a study on tiliapia (*Sarotherodon mossambicus*) showed that twins that were connected at their ventral part to the same yolk sac were connected at their abdomens as the yolk sac got absorbed [14]. In order to detect possible vascular connections early in life before the cardiovascular morphogenesis is completed, we conducted a follow-up study on Atlantic salmon conjoined twins that were connected at their ventral part to the same yolk sac.

## Results

### Twinning rate and phenotypes at hatch

**Fig. 1:**
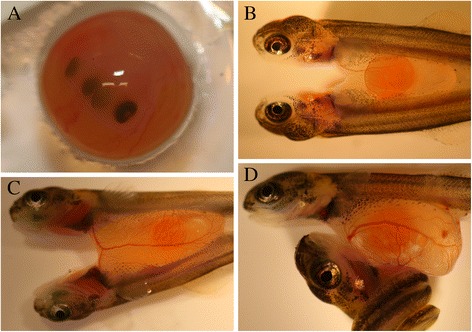
**a** An Atlantic salmon egg with conjoined twins. Note the presence of four eyes. It was not registered which type of twinning these individuals developed. **b**–**d** Photographs of ventrally conjoined twins of Atlantic salmon at the yolk sac larval stage. **b** Larvae without a distinct vascular connection. **c** Larvae with a vascular connection through their *v. vitellina hepatica.* **d** Larvae with some degree of vascular connection through their *v. vitellina hepatica*

**Fig. 2:**
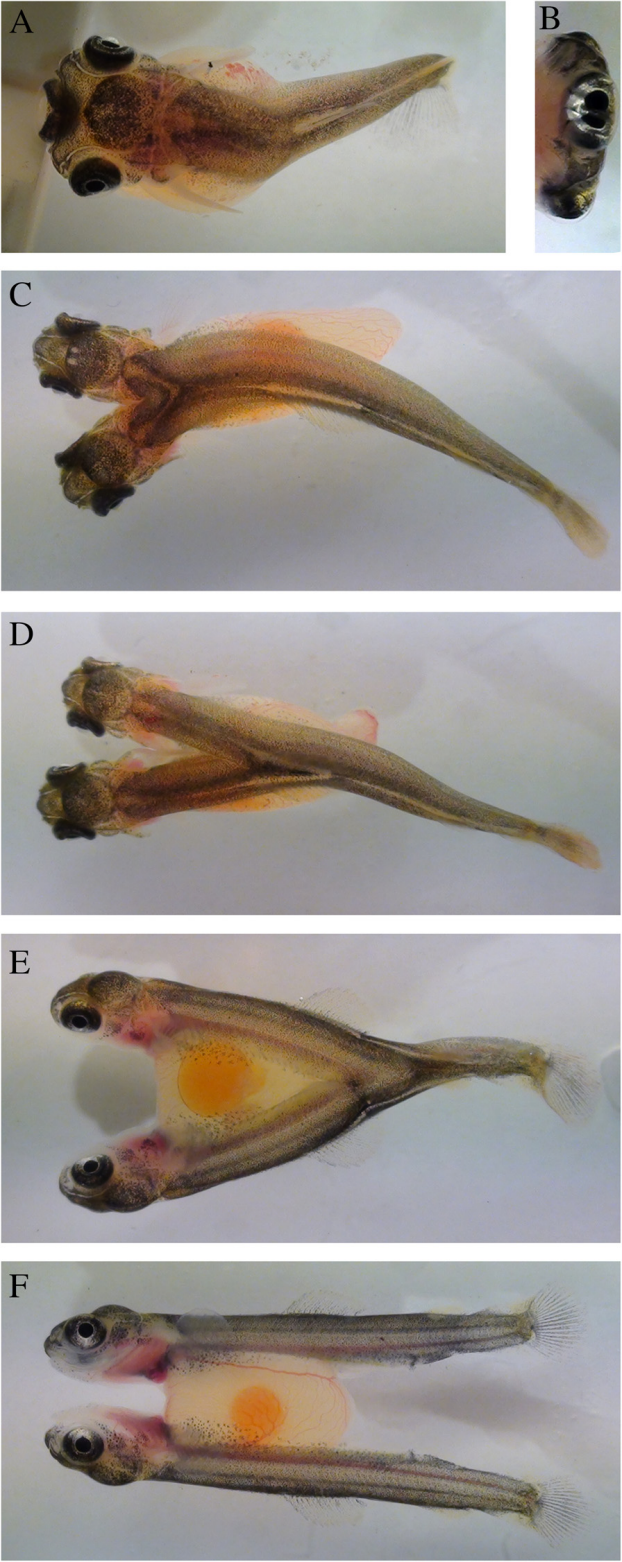
Pictures of different phenotypes of Atlantic salmon yolk sac larvae conjoined twins. **a** Twins laterally joined with a common large head with 4 eyes. **b** Front view of the specimen show in panel **a**. **c** Twins with two heads, and laterally joined by a common trunk and tail. **d** Twins laterally joined by a common posterior trunk and tail. **e** Twins laterally joined by a common tail. **f** Twins ventrally joined by a common yolk sac

Out of ~27 000 screened Atlantic salmon eggs, 120 eggs (0.4 %) with four eyes (Fig. 1a) were detected. Of these, 108 hatched (90 %), all conjoined twins or twins sharing a common yolk sac. These were categorized into five distinct phenotypes: (*i*) twins laterally joined with a common large head with four eyes (*n* = 20) (Fig. 2a,b); (*ii*) twins with two heads and laterally joined by a common trunk and tail (*n* = 33) (Fig. 2c); (*iii*) twins laterally joined by a common posterior trunk and tail (*n* = 3) (Fig. 2d); (*iv*) twins laterally joined by a common tail (*n* = 29) (Fig. 2e); (*v*) twins sharing a common yolk sac (*n* = 23) (Fig. 2f).

### Vascular connections early in life in twins that share a common yolk sac

Twins that were connected to the same yolk sac (type *v*), showed either no distinct cardiovascular connection (Fig. 1b), or different degrees of cardiovascular connections (Fig. 1c,d). The twin pair shown in Fig. 1c had a clearly joined *v. vitellina hepatica.* Inspection of the blood stream in a dissection binocular showed that blood cells were moving between the individuals through their joint vessel in the individual shown in Fig. 1c (data not shown).

### Anatomy and histology of a pair of fully developed ventrally conjoined twins

**Fig. 3:**
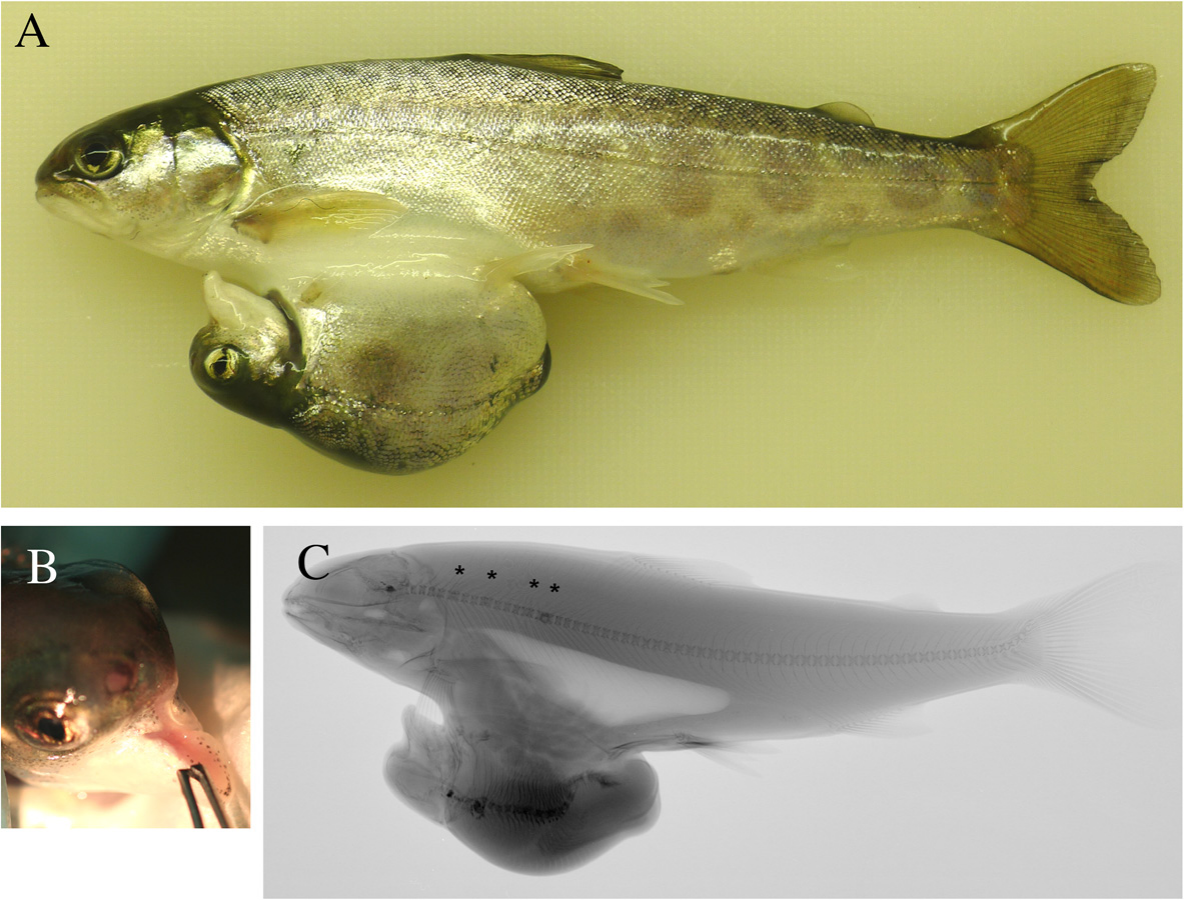
**a** Picture of Atlantic salmon x Arctic char hybrid twins. The upper twin is denoted ‘large twin’ and the lower ‘small’ twin in the text. **b** The mouth of the small twin was blocked. **c** Lateral radiograph. The large twin had 11 fused (asterix) vertebrae, while the small had severe cranial and vertebral deformities

The Atlantic salmon (♀) x Arctic char (♂) hybrid conjoined twins were attached by their abdomen (class *v*). One twin was of normal size and external anatomy, while the other twin was severely deformed and under-developed. When the twins were alive the large twin was swimming with the small twin hanging up-side down underneath (Fig. 3a). The length of the large twin was 14 cm, the length of the small 5 cm, and their total weight was 45.5 g. Externally, the small twin had the following abnormalities: blocked mouth (Fig. 3b), abnormal left eye, pugheadedness, downward curved lower jaw, left pelvic fin lacking, and small pectoral fins. The radiological examination showed that the vertebral column of the large twin had 11 fused vertebrae in the abdominal region, while the vertebral column of the small twin was severely deformed with large areas of fused vertebrae, and a curved and twisted vertebral column (Fig. 3c).

**Fig. 4:**
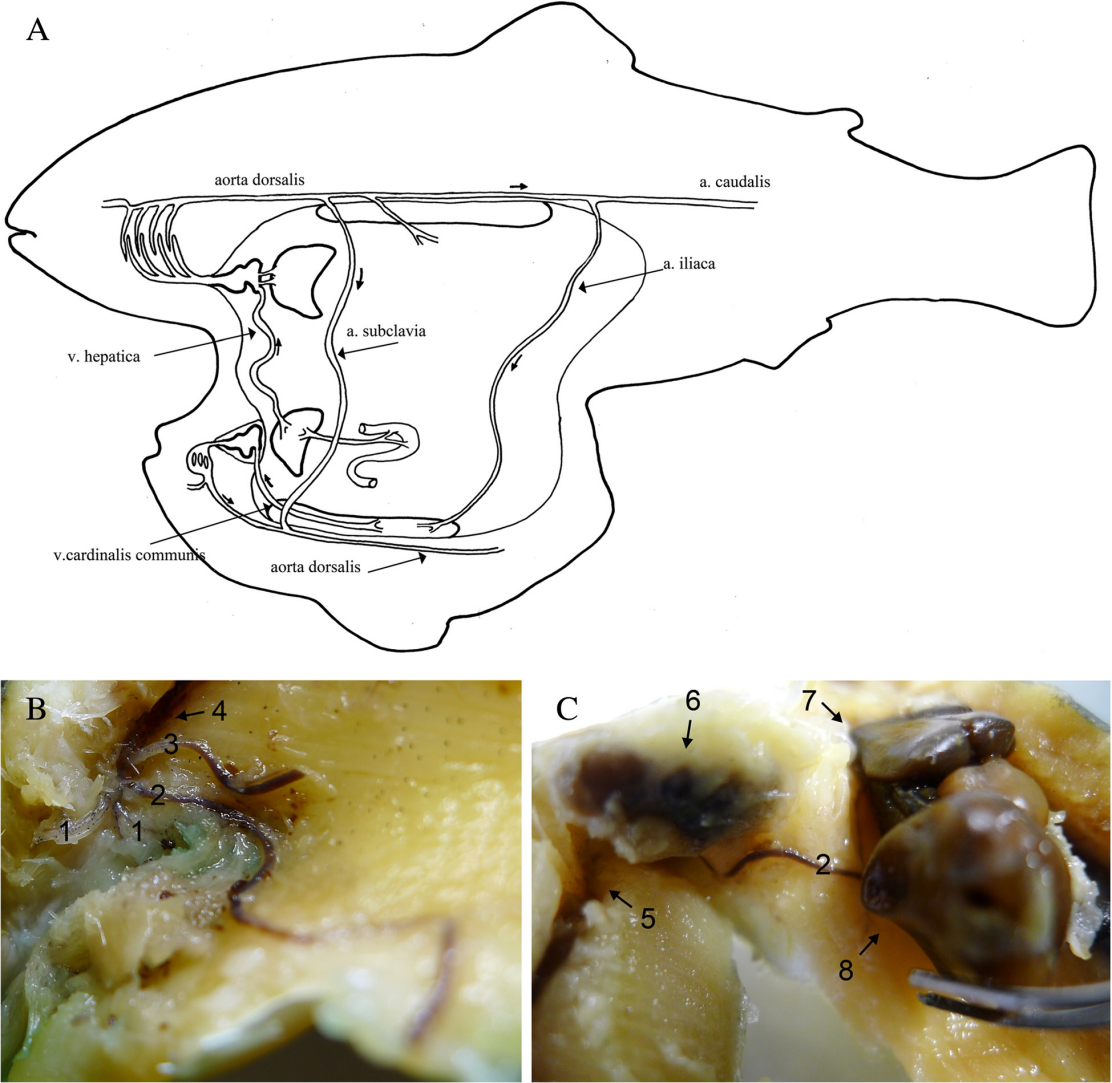
**a** Schematic drawing of the joint circulatory system between Atlantic salmon x Arctic char conjoined twins. **b** and **c** Photos taken during the dissection. (1) efferent arteries from the gill arches of the large twin. (2) *aorta subclavica* connecting the *aorta dorsalis* of the large twin (4) with the *aorta dorsalis* of the small twin (5). (3) *aorta coeliaca* of the large twin. (6) heart of the small twin covered by *septum transversum*. (7) heart of the large twin lacking *septum transversum*. (8) liver of the large twin

Internally, the twins had a common abdominal cavity, with all internal organs present in both twins. They were both females. The large and ‘normal’ twin lacked *septum transversum*, while the small deformed twin did not. Both twins had their separate hearts; these were connected through a common circulatory system (Fig. 4a). They were joined by the following blood vessels: (i) *arteria iliaca* running from *arteria caudalis* of the large twin to the kidney of the small twin; (ii) *arteria subclavia* running from *aorta dorsalis* of the large twin to *aorta dorsalis* of the small twin (Fig. 4b); (iii) *vena hepatica* running from the liver of the small twin into the *sinus venosus* of the large twin.

**Fig. 5:**
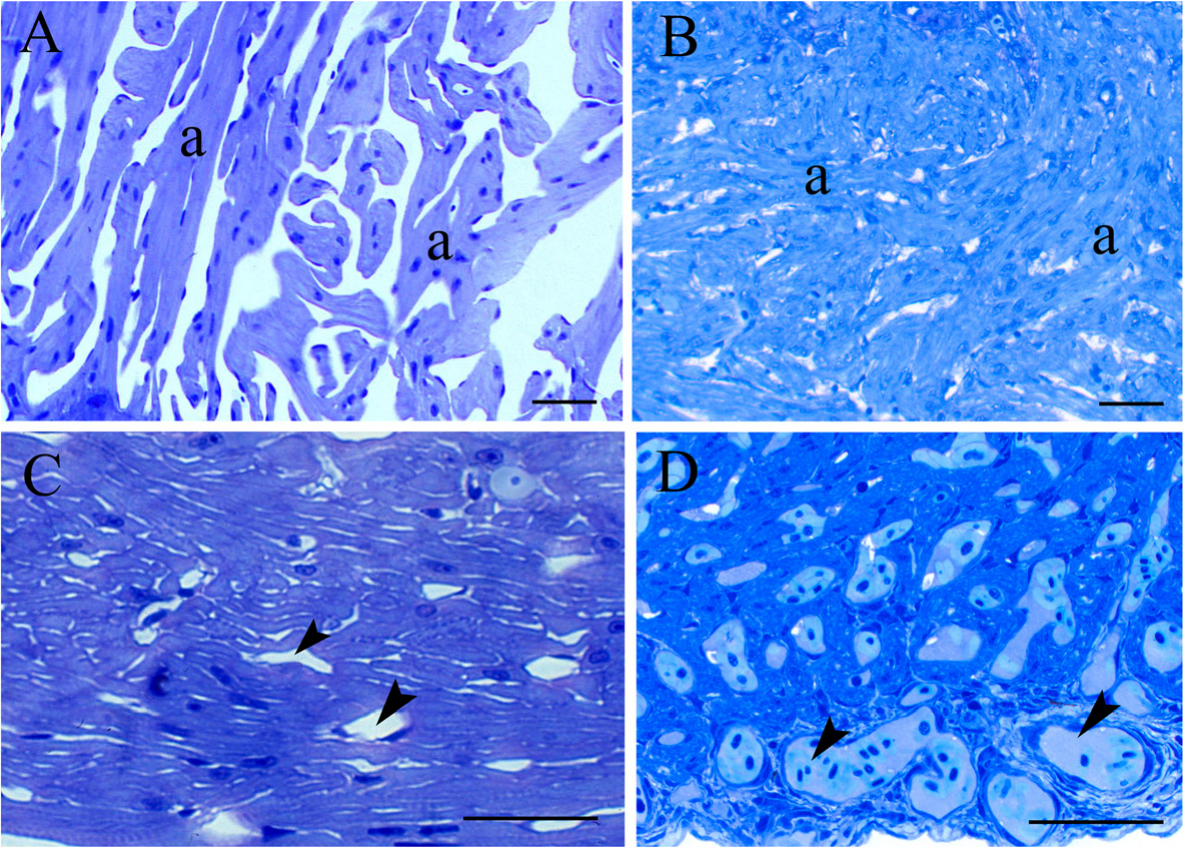
Representative photomicrographs of the heart structure. **a** and **c** are normal controls; **b** and **d** are from the minor twin. **a** and **b**: From the spongiosum, with trabeculae (*a*); notice how they are very well developed in **b**. **c** and **d** show the compactum. The capillaries (*arrowheads*) are large and numerous in **d**. Bar is 50 μm in all figures

**Fig. 6:**
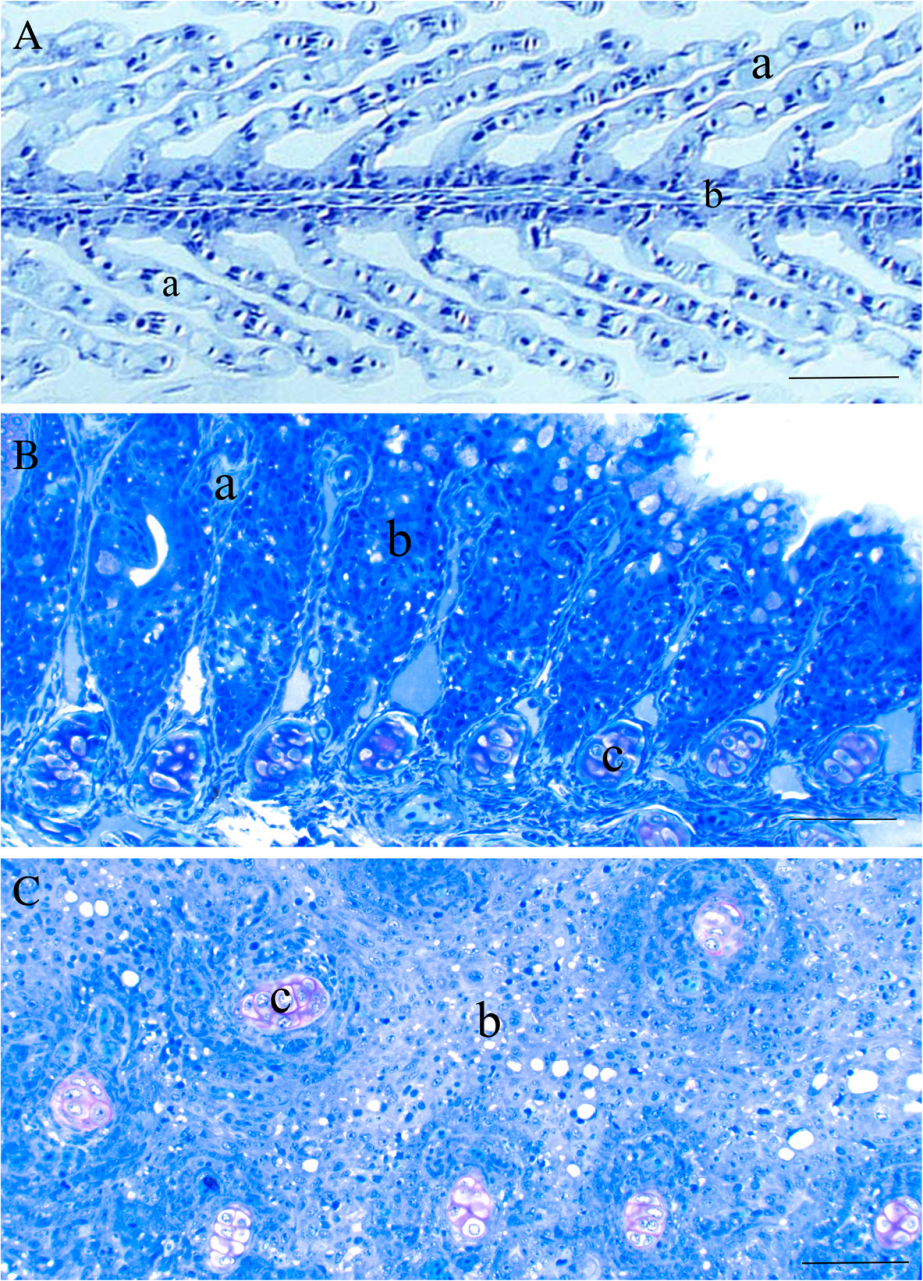
Gill anatomy. **a**: Normal control, with part of a gill filament (*b*); and numerous slender gill lamellae (*a*). **b**: Part of a gill structure from the smaller twin, with barely recognizable gill lamellae (*a*); surrounded by extensive hyperplastic epithelium (*b*). Here, a regular array of cartilage spicules is included (*c*). **c**: An irregular collection of cartilage spicules (*c*); surrounded by hyperplastic epithelium (*b*). Gill lamellae are not identifiable here. Bar is 50 μm in all pictures

**Fig. 7:**
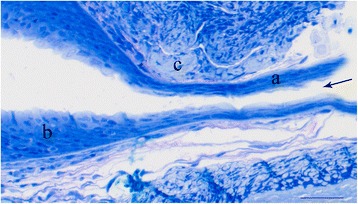
Photomicrograph of the very narrow mouth passage (*arrow*). Notice the low epithelium, *a*; indicating limited wear and tear. In the mouth cavity the epithelium has a normal height, *b*. The surrounding connective tissue is of normal composition, *c*. Bar is 50 μm

In general the histology of the larger twin was normal. In the smaller twin, the following studied tissues had also a normal appearance: the skin, the bones in the skeleton, and the digestive system. Histologically, the heart of the small twin also had a normal structure, but appeared to be hypertrophied; there was a densely developed spongiosum (Fig. 5b), and an abundance of capillaries in the musculature of the very well developed compactum (Fig. 5d). Normal control heart histology is shown in Fig. 5a and c. The gills of the small twin were small and under developed, the secondary lamellae were completely fused, due to massive hypertrophia of the gill epithelium. Only very few secondary lamellae showed normal structure; mostly lamellae were difficult to identify as such, more or less buried in the thick epithelium (Fig. 6b). In addition, the cartilaginous skeleton of the gills was arranged in a random fashion (Fig. 6c). Thus, due to the massive webbing of the secondary lamellae, the gills showed no signs of functionality. Normal control gill histology is shown in Fig. 6a. The passage through the mouth was very narrow (Fig. 7), with low squamous epithelium, and allowed neither normal feeding nor respiration. The pharynx was very short, had simple cylindric epithelium, and was fused into the stomach. Due to this, pharyngeal teeth were observed in the stomach wall. The pancreas and the mid- and hindgut had normal histological appearance, and the lumen of the stomach and intestines mainly filled with mucus. In the liver, the histology was normal, but melanomachophages were frequently seen.

### Conjoined twins are genetically identical

Two sets of type *v* Atlantic salmon yolk sac larvae twins were genotyped. Of the 18 microsatellite loci, 17 of them gave reliable genotypes in both sets of twins, while one failed to amplify in one of the sets. All informative microsatellite loci gave genotypes that were identical between the sets of twins, thus demonstrating that they were identical (Additional file 1).

Of the 109 SNP loci genotyped for the F1 salmon x char hybrid twins, 72 of them gave reliable genotypes in both of the twin samples, two gave unreliable clustering, while the remaining loci failed to amplify on some or all of the samples and where therefore non-informative (Additional file 1). According to the 72 loci that were successfully amplified in both twins, all gave identical genotypes, demonstrating that they were genetically identical.

## Discussion

This study shows that conjoined fish twins can have a joined cardiovascular system, and that one twin can support its conjoined sibling with both oxygen and nutrients. The fact that these blood vessel connections are visible also in yolk sac larvae shows that twinning in fish can be a valuable model in which to investigate cardiovascular morphogenesis in conjoined twins. This may contribute to fill in gaps in knowledge on this subject in humans, and support the development of best practice protocols for the treatment of human conjoined twins.

The yolk sac in fish is transparent, and the cardiovascular system and blood stream can easily be studied in vivo over time in the same individual through the yolk sac larvae stage. The time from hatching until the yolk sac is absorbed in Atlantic salmon is approximately 400 day degrees, meaning 67 days at 6 °C. Indeed, the heart rate in conjoined *Oreochromis mossambicus* twins have been successfully studied from day 2 to 8 after hatching [15]. Several different techniques can be applied to induce twinning in fish eggs, such as elevated water temperature [16, 17], hypoxia [18], irradiation, gynogenetic inbreeding [19], induction of mutations [20], and centrifugation (reviewed in [1]). That we have observed only one pair of conjoined twins that have survived the first feeding period in our research facility during the last 35 years, and that we found 0.4 % eyed eggs with four eyes in a random population of Atlantic salmon, all hatching to become conjoined twins, supports the notion that conjoined fish twins are rare and very seldom survive the transition from yolk sac absorbtion to external feeding. Previous studies have shown the occurrence of conjoined twins at hatch to be 0.2 % in seahorses (*Hippocampus guttulatus*) [21], 0.07 % in pink salmon (*Oncorhynchus gorbuscha*) [22], 0.6 % in Arctic char [23], 0.1 % in Nile tilapia (*Oreochromis niloticus*) and blue tilapia (*Oreochromis aureus*) [24], 0.3 % in *Porichthys notatus* [25], and 0.05 % in coho salmon (*Oncorhynchus kisutch*) [26]. Although the above mentioned literature shows that twinning is not a typical observation in fish fry, there are reports on massive outbreaks; for instance there is a report on an epidemia where only 10.000 out of 153.000 eggs put down survived, and the number of conjoined twins was observed to run into the thousands [16]. This was attributed to a temperature rise in the rearing water, and/or iron contamination. Also, a twinning rate ranging between 0.5 and 4 % was reported in a study where chum salmon (*Oncorhynchus keta*) eggs were incubated at 18 °C, in contrast to no twins at 8 °C [17]. It is possible that we could have kept some of the specimens studied herein alive through first feeding if special care was taken, and the rearing environment adjusted as such.

Conjoined twins of brook trout (*Salvelinus fontinalis*) have shown to develop *situs inversus viscerum* – symmetry reversal of the viscera [27]. Indeed, in humans, vascular anomalies such as presence of anomalous vessels have been reported in a case study on a cadaver with *situs inversus* [28]. However, whether the development of anomalous vessels that connect the cardiovascular systems of conjoined salmonid twins is related to the phenomena of *situs inversus* is not known. *Situs inversus* in fish conjoined twins may mean that the *v. vitellina hepaticas* are closely located and can grow into each other and fuse.

The twins investigated in the present study had intact circulatory systems, inter-connected by arteries and veins. This is the first time that this phenomena has been recorded in fish, most probably since conjoined twins rarely survive past the first feeding period, after the yolk sac is absorbed. There are a few records of larger specimens of conjoined twins, always pairs with one normal individual and one parasitic individual [3–6]. The smallest of the twins studied in the present study, with a blocked mouth and degraded and non-functional gills can also be regarded as parasitic.

The fully developed conjoined twin specimen dissected in the present study originated from a first-generation population of Atlantic salmon (♀) x Arctic char (♂) hybrids that had been held at a water temperature of 10 °C during egg incubation. In Atlantic salmon, 10 °C during egg incubation is high enough to induce developmental anomalies such as aplasia of *septum transversum*, and 8 °C is recommended as a maximum for commercial farming [29]. Earlier studies have shown that elevated temperature may [17], or may not [23] increase the occurrence of conjoined twins in salmonids. Further, heat shock treatment for 3–4 min applied 27 min after fertilization increased the twinning rate by three or four times over that of un-shocked eggs in Nile and blue tilapia [24]. It has been suggested – in salmonids – that elevated temperature results in the accumulation of unbroken cortical vesicles that disturbs the very early stages of development [17]. There are two specific causes of twinning in fish: *i*) early splitting of the blastodisc due to reduced cell adhesion during the early cell cycles [20]; (*ii*) effects on microtubule rearrangements leading to the aberrant transport of the dorsal determinant [30].

Although several studies of fish conjoined twins have performed detailed studies on tissue organization and histology [4–6, 21, 26], the present study is the first on the circulatory system in fully developed fish conjoined twins. Despite the fact that the small twin in the present study expressed anomalies in most body components and tissues, the digestive tract appeared normal both with regard to gross morphology and histology. Similarly, a parasitic salmonid twin that had grown into the body wall of a normal ‘host’ twin had a normal digestive tract [3]. Here, the other body parts of the parasitic twin were largely degenerated. Indeed, developmental fate maps show that the endoderm is derived from cells that are more vegetally located in the blastula and which may join the dorsal axis at a later developmental stage [31, 32], when cells may have had a chance of converging into a single tissue (even if induced in separate locations) during gastrulation. This could explain why endodermally-derived tissues are not affected during twinning.

The specimen that was dissected in the present study had a joined circulatory system but with separate hearts. An early study on the circulatory system in conjoined human twins [7] reported several different circulatory system phenotypes in twins with a joint circulatory system, some with one heart, or with two separate hearts, and some with ‘separate’ hearts joint through a common atrium or a transverse sac. A more recent study on the cardiovascular system of five cases of conjoined human twins [9] concluded that cardiac morphogenesis in conjoined twins appears to depend on the site of the conjoined fusion. In humans, the arrangement of the cardiovascular system is largely dependent on the external morphological type [9, 33], where 75 % of thoracopagus – the most common morphological type and joined by the thorax – conjoined twins have a fused heart [34]. All the 5 types of conjoined twins defined herein have similar human phenotypes [35]. The yolk sac larvae conjoined twins in the present study showed a large variation in where along the body axis they were fused, and some only shared a common yolk sac. How twinning type relates to the arrangement of the cardiovascular system in conjoined fish twins needs to be studied in more detail in order to evaluate the suitability of fish conjoined twins as model organisms to study cardiovascular morphogenesis in conjoined twins. The different level of attachment in the herein observed conjoined twins may depend on the stage of development when the secondary fusion occurs. The attachment point will most probably move caudally with increasing time between primary fission and secondary fusion [36], meaning that the types *i* to *iv* classified herein may reflect increasing time between primary fission and secondary fusion. Unfortunately we did not genotype these types. However, the genotyping of the type *v* confirms that these originated from a primary fission of the early cleaving blastoderm. In the herein type *v*, the embryos do not fuse macroscopically, but develops into two separate individuals that undergoes ‘secondary fusion’ after the yolk sac is absorbed. However, their cardiovascular systems may be fused. In Atlantic salmon, the vitelline vein begins to spread over the yolk sac at the end of somitogenesis [36], making a fusion of the cardiovascular system after somitogenesis is completed possible in twins that share a common yolk sac. Whether this path of abnormal development (type *v* herein) is developmentally similar to the normal development of un-conjoined monozygotic twins in humans, is unclear. Suggesting this, is a record of 144 and 146 mm long twin embryos of Shortnose spurdog (*Squalus megalops*), only conjoined by a 36 mm long and 1.5 mm thick cord of embryonic tissue [37]. The point of attachment to the yolk sac in sharks is indeed much smaller compared to that in salmonids.

The small parasitic and handicapped twin studied herein would have died without the support of oxygen and nutrients from its bigger host twin. Death of the small twin would most probably be detrimental for the large twin. Hence, the development of a joined circulatory system may have been a mechanism to support life, or it may just be a developmental anomaly.

## Conclusions

This study demonstrates that ventrally conjoined fish twins can develop cardiovascular connections during early development, and that this can enable a normal superior twin to supply a malfunctioning twin with oxygen and nutrients. Since the yolk sac is transparent and the cardiovascular system can be inspected in vivo in the period from hatching until the fish starts feeding, twinning in fish can serve as a model to understand the basis of the cardiovascular morphogenesis in conjoined twins.

## Methods

### Dissection of a pair of fully developed conjoined twins

The specimens used in this study originated from a first-generation population of Atlantic salmon (♀) x Arctic char (♂) hybrids, which were fertilized on 16 December 2010. The ova originated from a mix of five salmon females from the domesticated AquaGen strain, and the sperm was from three wild-caught Arctic char from Hopsvatnet Lake in western Norway. The hybrid population was reared under conditions normally used for Atlantic salmon aquaculture, and the studied conjoined twin was sampled 1 year after fertilization in December 2011. The twins were euthanized with an over-dose of anesthetics (Finquel®, ScanAqua).

After the fork length of the largest twin and the total body weight of both twins had been measured, they were radiographed. Thereafter, the twins were carefully dissected to reveal the internal organs organization. After the location of heart, liver, spleen, digestive tract, swim bladder and kidney were assessed in both individuals; they were put in 4 % formalin for fixation. After the fixation was completed the complete anatomy of the circulatory system was described by careful blunt dissection.

### Histology

For analyses using the microscope, the following organs from both individuals were dehydrated in 96 % ethanol, embedded in Technovit, and finally sectioned at 2 microns: heart, liver, gills, skin, stomach and mid-gut. Sections were stained in Toluidine blue and studied and photographed in an Olympus Vanox microscope.

### Screening for yolk sac larvae conjoined twins

The twins we dissected were connected at their abdomens. When these twins hatched, they were most probably connected at their ventral part to the same yolk sac and got connected at their abdomens as the yolk sac got absorbed, as shown in tilapia (Hulata and Rothbard, 1978). In order to study the blood vessels in the yolk sac of twins that are connected at their ventral part to the same yolk sac, we screened Atlantic salmon eyed eggs for the presence of four eyes (Fig. 1a). The Atlantic salmon twins originated from separate families of the domesticated Mowi strain, produced at Matre Research station, on 12 December, 2014.

These eggs were hatched, and yolk sac larvae that were ventrally conjoined had their common yolk sac investigated for possible vascular connection. The investigation was done prior to the onset of exogenous feeding, and the yolk sac larvae were sedated (0.07 gL^−1^, Finquel®, ScanAqua) during inspection, and immediately euthanized afterwards with an over-dose of anesthetic (0.4 gL^−1^, Finquel®, ScanAqua). The inspection was done under a dissection binocular. Also, we classified all twins obtained from the screening into 5 classes according to their external anatomy.

### Genotyping

Ethanol-preserved tissue samples from two sets of ventrally conjoined Atlantic salmon twins (Fig. 1c,d) were subjected to a standard DNA isolation using a commercially available kit. Thereafter, 18 highly polymorphic microsatellite loci were amplified and genotyped on an ABI3730XL DNA sequencer. Finally, the allelic profiles of these samples were scored using GeneMapper V5.0 and thereafter compared to each other in order to identify if they were genetically identical or not. More extensive details with respect to the exact genotyping procedure are available elsewhere [38].

In contrast to the Atlantic salmon twin samples which had been preserved in ethanol, the salmon x char hybrid twin had been fixed in formalin prior to any genetic analysis. Formalin provides challenges to extract high quality DNA, and the standard DNA isolation method as implemented for the Atlantic salmon twins above was unsuccessful. Consequently, tissue samples from the salmon x char twin were sent to the company Eurofins in Germany that operate a forensic genetics laboratory certified for amongst other things DNA isolations according to ISO 17025. Here, a more comprehensive DNA extraction targeted at degraded tissues was conducted. The extracted DNA was thereafter returned to IMR for genotyping. The quality of the DNA resulting from this extraction was still insufficient for microsatellite DNA analysis using the same Atlantic salmon markers as described above, and therefore, these samples were genotyped on a Sequenom MassARRAY system for 109 bi-allelic SNPs isolated from Atlantic salmon that were distributed throughout the genome [39].

## Abbreviations

SNP, single nucleotide polymorphism

## Additional file

Additional file 1: Genetic identification of the salmon-char twins using 109 biallelic SNPs isolated from Atlantic salmon, and Genetic identification of the Atlantic salmon twins using 18 polymorphic microsatellite DNA loci. (PDF 249 kb)
