# Supplementary material for: Salmonid fish: model organisms to study cardiovascular morphogenesis in conjoined twins?
Source: BMC Dev Biol. 2016 Jul 16;16:25. doi: 10.1186/s12861-016-0125-x (PMC4947332; doi:10.1186/s12861-016-0125-x)
Supplement: Additional file 1: — Genetic identification of the salmon-char twins using 109 biallelic SNPs isolated from Atlantic salmon, and Genetic identification of the Atlantic salmon twins using 18 polymorphic microsatellite DNA loci. (PDF 249 kb) [file 12861_2016_125_MOESM1_ESM.pdf]

SUPPLEMENTAL MATERIAL

Genetic identification of the char-salmon twins using 109 biallelic SNPs isolated from Atlantic salmon.

Blank cell = no marker amplification.

Genetic markers correspond with Lien et al., 2011 genetic map.

Markers giving unreliable genotyping are marked with comment

Total number of usable markers = Ok = 72

Total number of usable markers with identical genotype junior and senior (72 = 100%)

Genetic marker	Junior muscle	Senior Liver	Senior Muscle	Senior Heart	Comment
BASS117_B7_F06_677	CC	CC	CC	CC	OK
ESTNV_20218_62	CC	CC	CC	CC	OK
ESTNV_21396_116	CC	CC	CC	CC	OK
ESTNV_25066_386	TT	TT	TT	TT	OK
ESTNV_25207_614	CC	CC	CC	CC	OK
ESTNV_31779_190	CT	CT	CT	CT	OK
ESTNV_33205_897	AG		AG		OK
ESTNV_35065_572	CC	CC	CC	CC	OK
ESTNV_36651_1364	GG	GG		GG	OK
ESTV_18140_753	CC	CC	CC	CC	OK
ESTV_20442_1276	CC	CC	CC	CC	OK
GCR_cBin10506_Ctg1_377	AA	AA	AA	AA	OK
GCR_cBin10764_Ctg1_187	AA	AA		AA	OK
GCR_cBin15290_Ctg1_216	CC	CC	CC	CC	OK
GCR_cBin1608_Ctg1_208	CT	CT	CT	CT	OK
GCR_cBin207_Ctg1_149	AA	AA	AA	AA	OK
GCR_cBin20876_Ctg1_29	CC	CC	CC	CC	OK
GCR_cBin25731_Ctg1_87			CC		Poor amplification
GCR_cBin30435_Ctg1_161					Poor amplification
GCR_cBin39106_Ctg1_120	TT	TT	TT	TT	OK
GCR_cBin40838_Ctg1_52	AA	AA	AA	AA	OK
GCR_cBin49296_Ctg1_187	GG	GG	GG	GG	OK

GCR_cBin49589_Ctg1_81	TT	TT	TT	TT	OK
GCR_cBin501_Ctg1_254					Poor amplification
GCR_cBin7278_Ctg1_140	GG	GG	GG	GG	OK
GCR_hBin1984_Ctg1_131	AC	AC	AC	AC	OK
BASS14_B7_H10_429	CC	CC	CC	CC	OK
ESTNV_22696_678	TT	TT	TT	TT	OK
ESTNV_23861_220	CC	CC	CC	CC	OK
ESTNV_29410_233	CC	CC	CC	CC	OK
ESTNV_30996_234	GG	GG	GG	GG	OK
ESTNV_33016_564	AA	AA		AA	OK
ESTNV_33128_565	AA	AA	AA	AA	OK
ESTNV_36072_124	GG	GG	GG	GG	OK
ESTV_14782_767	GG	GG		GG	OK
ESTV_16103_649					Poor amplification
ESTV_16688_280	AG	AG	AG	AG	OK
ESTV_16913_418	TT	TT	TT	TT	OK
GCR_cBin13730_Ctg1_588					Poor amplification
GCR_cBin14803_Ctg1_153	AA	AA	AA	AA	OK
GCR_cBin18802_Ctg1_133	TT	TT	TT	TT	OK
GCR_cBin19498_Ctg1_386	AA		AA	AA	OK
GCR_cBin2349_Ctg1_433	CG	CG		CG	OK
GCR_cBin2393_Ctg1_311	TT	TT	TT	TT	OK
GCR_cBin25356_Ctg1_418	AA	AA	AA	AA	OK
GCR_cBin27805_Ctg1_63		GT			Poor amplification
GCR_cBin34406_Ctg1_88	CC	CC		CC	OK
GCR_cBin36544_Ctg1_139	CC	CC	CC	CC	OK
GCR_cBin39236_Ctg1_56	GG	GG		GG	OK
GCR_cBin466_Ctg1_234	GG	GG	GG	GG	OK
GCR_cBin5664_Ctg1_209	CC	CC	CC	CC	OK
GCR_cBin6507_Ctg1_263	AA	AA	AA	AA	OK
GCR_cBin760_Ctg1_479					Poor amplification
GCR_cBin774_Ctg1_544	GG	GG	GG	GG	OK
GCR_cBin8161_Ctg1_218	GG	GG	GG	GG	OK

GCR_cBin9358_Ctg1_120	AA	AA	AA	AA	OK
GCR_hBin27289_Ctg1_90	TT	TT	TT	TT	OK
ESTNV_21223_122	TT	TT	TT	TT	OK
ESTNV_22734_289	CC	CC	CC	CC	OK
ESTNV_29299_371					Poor amplification
ESTNV_32516_218	CC	CC	CC	CC	OK
ESTNV_33917_998					Poor amplification
ESTNV_34451_135	AA	AA	AA	AA	OK
ESTNV_34935_1675	CC	CC	CC	CC	OK
GCR_cBin10758_Ctg1_395					Poor amplification
GCR_cBin10801_Ctg1_300			TT		Poor amplification
GCR_cBin11286_Ctg1_158	GG	GG	GG	GG	OK
GCR_cBin11714_Ctg1_79	CT	CT	CT	CT	OK
GCR_cBin1188_Ctg1_179	AA	AA	AC	AA	Unreliable clustering
GCR_cBin17416_Ctg1_223	TT	TT	TT	TT	OK
GCR_cBin23187_Ctg1_181	GG	GG	GG	GG	OK
GCR_cBin24202_Ctg1_90	CC	CC	CC	CC	OK
GCR_cBin25832_Ctg1_468	GG	GG	GG	GG	OK
GCR_cBin32185_Ctg1_72	AA	AA	AA	AA	OK
GCR_cBin33206_Ctg1_109	TT	TT	TT	TT	OK
GCR_cBin41047_Ctg1_75	GG	GG	GG	GG	OK
GCR_cBin43612_Ctg1_147	CC	CC	CC	CC	OK
GCR_cBin4585_Ctg1_148	AA	AA	AA	AA	OK
GCR_cBin48621_Ctg1_146			CT		Poor amplification
GCR_cBin4944_Ctg1_147	CC	CC	CC	CC	OK
GCR_cBin50954_Ctg1_86	GG	GG	GG	GG	OK
GCR_cBin51554_Ctg1_52	TT	TT	TT	TT	OK
GCR_cBin5440_Ctg1_39	AA	AA	AA	AA	OK
GCR_cBin6469_Ctg1_121	AA	AA	AA	AA	OK
GCR_hBin16337_Ctg1_198	GG	GG	GG	GG	OK
ESTNV_16657_595		AA	AA	AA	Poor amplification
ESTNV_23285_157		CC	CC	CC	Poor amplification
ESTNV_28366_1826		GG	GG	GG	Poor amplification

ESTNV_30562_773		CT	CT	CT	Poor amplification
ESTNV_33266_651					Poor amplification
ESTNV_34453_617		AA	AA	AA	Poor amplification
ESTNV_34663_147					Poor amplification
ESTNV_35432_2607		AA		AA	Poor amplification
ESTNV_36751_1595		CC	CC	CC	Poor amplification
ESTNV_36856_820		AA	AA	AA	Poor amplification
ESTNV_37138_2049		TT	TT	TT	Poor amplification
ESTV_16609_285		GG		GG	Poor amplification
ESTV_16691_375		CC	CC	CC	Poor amplification
ESTV_17632_161					Poor amplification
GCR_cBin12293_Ctg1_225		TT	TT	TT	Poor amplification
GCR_cBin13678_Ctg1_110	AA	TT	AT	TT	Unreliable clustering
GCR_cBin14116_Ctg1_119		GG	GG	GG	Poor amplification
GCR_cBin28606_Ctg1_136		GG	GG	GG	Poor amplification
GCR_cBin33614_Ctg1_90		GG	GG	GG	Poor amplification
GCR_cBin34474_Ctg1_164		CC	CC	CC	Poor amplification
GCR_cBin37713_Ctg1_64		CC		CC	Poor amplification
GCR_cBin38161_Ctg1_138		GG	GG	GG	Poor amplification
GCR_cBin44616_Ctg1_312		CC	CC	CC	Poor amplification
GCR_cBin6366_Ctg1_223		TT	TT	TT	Poor amplification

Genetic identification of the Atlantic salmon twins using 18 polymorphic microsatellite DNA loci

Blank cell = no marker amplification.
Total number of usable markers with identical genotype siamese twin 1: 17 (100% similar)
Total number of usable markers with identical genotype siamese twin 2: 18 (100% similar)

Sample	Siamese twins 1a (34-21)	Siamese twin 1b (34-22)	Siamese twin 2a (39-2)	Siamese twin 2b (39-3)	Comment
MHC1-a	164	164	146	146	OK
MHC1-b	164	164	148	148	OK
MHC2-a	210	210	260	260	OK

MHC2-b	260	260	360	360	OK
SSsp2201-a	295	295	251	251	OK
SSsp2201-b	295	295	295	295	OK
SSsp2210-a	124	124	124	124	OK
SSsp2210-b	152	152	132	132	OK
SSsp3016-a	90	90	98	98	OK
SSsp3016-b	102	102	102	102	OK
SSspG7-a	127	127	143	143	OK
SSspG7-b	143	143	179	179	OK
Sp1605-a	224	224	224	224	OK
Sp1605-b	249	249	244	244	OK
Sp2216-a	222	222	226	226	OK
Sp2216-b	226	226	230	230	OK
SsOsl85-a	193	193	187	187	OK
SsOsl85-b	197	197	187	187	OK
Ssa14-a	140	140	140	140	OK
Ssa14-b	140	140	140	140	OK
Ssa171-a			217	217	Poor amplification
Ssa171-b			221	221	Poor amplification
Ssa197-a	176	176	180	180	OK
Ssa197-b	196	196	188	188	OK
Ssa202-a	242	242	238	238	OK
Ssa202-b	254	254	258	258	OK
Ssa289-a	112	112	112	112	OK
Ssa289-b	128	128	118	118	OK
SsaD144-a	162	162	122	122	OK
SsaD144-b	166	166	162	162	OK
SsaD157-a	343	343	311	311	OK
SsaD157-b	363	363	339	339	OK
SsaD486-a	172	172	172	172	OK
SsaD486-b	172	172	172	172	OK
SsaF43-a	115	115	103	103	OK
SsaF43-b	115	115	115	115	OK
